# Factors related to good asthma control using different medical adherence scales in Latvian asthma patients: an observational study

**DOI:** 10.1038/s41533-017-0042-x

**Published:** 2017-06-20

**Authors:** Dins Smits, Girts Brigis, Jana Pavare, Baiba Maurina, Noël Christopher Barengo

**Affiliations:** 1 Faculty of Public Health and Social Welfare, Riga Stradins University, Dzirciema 16, Riga, LV-1007 Latvia; 2 Faculty of Medicine, Riga Stradins University, Dzirciema 16, Riga, LV-1007 Latvia; 3 Faculty of Pharmacy, Riga Stradins University, Dzirciema 16, Riga, LV-1007 Latvia; 4 Herbert Wertheim College of Medicine, Florida International University, 11200 SW 8th Street, AHC1, Miami, FL 33199 USA

## Abstract

One of the main challenges in asthma control is adherence to pharmaceutical treatment. The aim of this study was to test the association between adherence to asthma medication, control and medical beliefs, cognitive and emotional perceptions using three different validated questionnaires. Beliefs about asthma medicine, cognitive and emotional factors were determined in a cross-sectional survey of patients attending outpatient pulmonologist practices in Latvia (*n* = 352). The validated Beliefs about Medicines Questionnaire and the Brief Illness Perception Questionnaire were used. Adherence to asthma medication was assessed using the Morisky Medication Adherence Scale and two different versions of the Medication Adherence Reporting Scale. Several questions about necessity or concerns related to pharmaceutical treatment were able to predict poor adherence according to the Morisky scale. If the patient felt that without the asthma medication his life would be impossible, his risk to have poor treatment adherence was 46% reduced (odds ratio 0.54; 95% confidence interval 0.33–0.89). Furthermore, asthma patients who were convinced that their health depends on the asthma treatment were less likely to have poor treatment adherence (odds ratio 0.56: 95% confidence interval 0.32–0.97). In case the patient was concerned by the need to constantly use asthma medication or sometimes concerned by long-term effects of asthma medication the odds of poor treatment adherence were 1.96 (95% confidence interval 1.19–3.24) and 2.43 (95% confidence interval 1.45–4.08), respectively. In conclusion, medication beliefs, particularly concerns and necessity of asthma treatment were associated with poor treatment adherence when assessed with the Morisky or 5-item Medication Adherence Reporting Scale.

## Asthma treatment: Investigating poor adherence in the Latvian population

Concerns about treatment necessity and uses of asthma medication are key factors influencing poor treatment adherence in Latvia. There are multiple psychological and behavioral reasons why patients do not adhere to asthma treatment courses, including social and religious beliefs, and perceptions of chronic illness. Patient questionnaires and ‘adherence scales’ can help predict whether patients are likely to follow advice, but their efficacy differs for individual nations. Dins Smits at Riga Stradins University, Latvia, and co-workers analyzed responses to three questionnaires completed by 352 asthma patients to assess treatment perceptions and the best questionnaire option for the Latvian population. Concerns about the use of asthma medication and beliefs about treatment necessity were key factors in poor adherence. These were picked up by the Morisky and MARS 5-item scales, which the authors recommend for future use.

## Introduction

Asthma is a serious global health problem affecting approximately 300 million people worldwide.^1, 2^ The estimated prevalence of people living with asthma ranges from 1 to 21% in adults and with up to 20% of children aged 6–7 years experiencing severe wheezing episodes within a year.^3, 4^

One of the main challenges in asthma control is adherence to pharmaceutical treatment. Generally, poor adherence is common across many chronic diseases such as asthma.^5, 6^ As it is not easy to test clinically for adherence, several questionnaires have been developed to assess adherence to medication.^7, 8^ These questionnaires are widely used in many countries. However, no information is available on treatment adherence of asthma patients in Latvia.

Several factors influence treatment adherence. Some of these factors are related to complexity of the therapy, fear of side effects of drugs, method of taking the drug, dosage regimen, adverse events, knowledge about the essence of the disease and its complications, illness perception and social support.^6, 9, 10^ Especially, medical beliefs, cognitive and emotional perceptions have shown to be related to medical adherence.^11, 12^ Furthermore, individual asthma management might be improved by a better understanding of the types of beliefs or illness-related perceptions that are associated with low adherence to preventer medication.^13^ Vrijens et al.’s proposed Ascertaining Barriers to Compliance (ABC) taxonomy conceptualizes adherence to medications in line with principles of behavioral and pharmacological science.^14^ The ABC taxonomy defines the overarching concept of “medication adherence” as the process by which patients take their medication as prescribed and subdivides it into 3 essential elements: (A) initiation; (B) implementation, and (C) persistence. This subdivision outlines the sequence of events that have to occur for a patient to experience the optimal benefit from his or her prescribed treatment regimen.^14^

Thus, comparing how well these factors predict treatment adherence in the currently available adherence scores, may give valuable insight in the questionnaire-of-choice for the Latvian asthma population in order to be implemented in clinical practice throughout the country.

The aim of this study was to test the association between adherence to asthma medication, control and medical beliefs, cognitive and emotional perceptions using three different validated questionnaires in Latvian asthma patients.

## Results

The baseline characteristics of the study population are presented in Table 1.

**Table 1:** Baseline characteristics of the study sample

	Men	Women	Total	
	(*n* = 85)	(*n* = 264)	(*n* = 352)	*p*-value
Age, mean (SD)	53.7 (17.4)	58.7 (16.6)	57.5 (16.9)	0.017
Education, %				0.006
Basic	3.5	7.5	6.5	
Secondary	23.3	27.1	26.1	
Professional	47.7	28.9	33.5	
Higher	25.6	36.5	33.8	
Income, %				0.005
<300 €/month	16.7	25.3	23.2	
300–550 €/month	36.9	48.2	45.5	
550–750 €/month	28.6	18.3	20.8	
>750 €/month	17.9	8.2	10.6	
Asthma medication, %				
Corticosteroids	62.8	63.4	63.3	1.00
Corticosteriods + beta 2 mimetic	33.7	33.2	33.3	1.00
Poor asthma control, %	62,8	66	65,3	0.604
Poor treatment adherence, %				
Morisky scale	72.1	69.4	70.1	0.686
MARS (5-item) scale	58.1	68.7	66.1	0.089
MARS (10-item) scale	75.6	69	70.6	0.278

The majority of the patients had professional education and were earning at least 300 euros per month. Two out of three patients were using corticosteroids and one third a combination therapy consisting of corticosteroids and a beta 2 mimetic drug. The prevalence of poor asthma control was 63% in men and 66% in women. The prevalence of poor treatment adherence ranged between 58–76% in men and 69% in women. Age, income, and education were statistically significantly differently distributed between men and women.

Table 2 shows the associations of the different asthma medication adherence scores with poor asthma control in the study sample. None of the three adherence scores was able to predict poor disease control in Latvian asthma patients.

**Table 2:** Associations of different asthma medication adherence scores with poor asthma control

	Univariate		Multivariate^a^	
	OR	(95% CI)	OR	(95% CI)
Men				
MARS (5-item)	0.39	(0.15–1.00)	0.36	(0.12–1.09)
MARS (10-item)	1.05	(0.38–2.90)	1.41	(0.43–4.65)
Morisky	1.02	(0.38–2.69)	1.48	(0.49–4.50)
Women				
MARS (5-item)	1.04	(0.60–1.79)	1.2	(0.67–2.17)
MARS (10-item)	1.68	(0.98–2.87)	1.61	(0.91–2.86)
Morisky	1.09	(0.63–1.89)	0.94	(0.52–1.70)
All				
MARS (5-item)	0.81	(0.51–1.30)	0.87	(0.52–1.44)
MARS (10-item)	1.5	(0.93–2.40)	1.43	(0.87–2.36)
Morisky	1.07	(0.67–1.72)	0.99	(0.60–1.66)

>6 (MARS-5), >14 (MARS-10), >1 (MMAS) was used to define poor medical adherence; ≤19 for the ACT was defined to indicate poorly controlled asthma

*OR* odds ratio, *CI* confidence interval

^a^ Adjusted for age, education and income

None of the socio-demographic or socio-economic factors were predictors of poor treatment adherence according to the Morisky or the MARS 10-item scale (Table 3). Further adjustment did not change the findings of the unadjusted model.

**Table 3:** Associations of socio-demographic and socio-economic factors with poor treatment adherence in asthma patients

	Morisky scale				MARS (5-item)				MARS (10-item)			
	Unadjusted		Adjusted^a^		Unadjusted		Adjusted		Unadjusted		Adjusted	
	OR	(95% CI)	OR	(95% CI)	OR	(95% CI)	OR	(95% CI)	OR	(95% CI)	OR	(95% CI)
Age	1	(0.98–1.01)	1	(0.98–1.01)	0.98	(0.97–1.00)	0.98	(0.96–0.99)	1	(0.99–1.02)	1	(0.98–1.01)
Female sex	0.88	(0.51–1.50)	0.86	(0.48–1.54)	1.58	(0.96–2.60)	1.87	(1.07–3.27)	0.72	(0.41–1.26)	0.65	(0.35–1.18)
Education												
Basic or secondary	1	Ref^b^	1	Ref	1	Ref	1	Ref	1	Ref	1	Ref
Professional	1.43	(0.82–2.51)	1.55	(0.86–2.78)	1.79	(1.03–3.11)	1.91	(01.05–3.45)	1.23	(0.69–2.17)	1.15	(0.63–2.08)
Higher	1.45	(0.83–2.53)	1.72	(0.92–3.22)	1.27	(0.75–2.16)	1.25	(0.68–2.29)	0.97	(0.56–1.69)	1.16	(0.62–2.17)
Income												
<300 €/month	1	Ref	1	Ref	1	Ref	1	Ref	1	Ref	1	Ref
300–550 €/month	0.83	(0.45–1.52)	0.74	(0.40–1.38)	0.86	(0.48–1.55)	0.89	(0.48–1.65)	0.86	(0.46–1.58)	0.81	(0.43–1.53)
550–750 €/month	0.67	(0.33–1.34)	0.51	(0.24–1.09)	0.8	(0.41–1.59)	0.66	(0.31–1.43)	0.62	(0.31–1.26)	0.54	(0.25–1.17)
>750 €/month	1.27	(0.50–3.22)	0.92	(0.33–2.54)	0.87	(0.38–2.03)	0.75	(0.29–1.93)	0.77	(0.32–1.84)	0.64	(0.25–1.69)
Asthma medication												
Corticosteroids	1.34	(0.83–2.16)	0.82	(0.49–1.36)	0.57	(0.35–0.91)	0.56	(0.33–0.93)	0.69	(0.42–1.12)	0.72	(0.43–1.21)
Corticosteriods + beta 2 mimetic	1.04	(0.64–1.69)	0.85	(0.51–1.41)	1.93	(1.18–3.16)	2.04	(1.19–3.49)	1.18	(0.72–1.93)	1.11	(0.66–1.86)

*OR* odds ratio, *CI* confidence interval

^a^ Adjusted for age, sex, education and income

^b^ Reference group

However, when the Medication Adherence Reporting Scale (MARS) 5-item scale was used, increasing age (odds ratio (OR) 0.98 (95% confidence interval (CI) 0.97–1.00) and monotherapy with corticosteroids (OR 0.57; 95% CI 0.35–0.91) reduced the odds of poor treatment adherence. Moreover, professional education level (OR 1.79; 95% CI 1.03–3.11) or the combined use of corticosteroids and beta 2 mimetics (OR 1.93; 95% CI 1.18–3.16) increased the odds of poor treatment adherence.

The associations of cognitive and emotional illness indicators and poor treatment adherence measured using three different scores in asthma patients in Latvia are presented in Table 4. None of the eight items of the brief Illness Perception Questionnaire (Brief IPQ) was a statistically significant predictor of poor treatment adherence in any of the three adherence scores. Adjustment for age, education or income did not alter the results.

**Table 4:** Associations of cognitive and emotional illness indicators and poor treatment adherence measured using three different scores in asthma patients in Latvia

	Morisky scale				MARS (5-item)				MARS (10-item)			
	Unadjusted		Adjusted^a^		Unadjusted		Adjusted		Unadjusted		Adjusted	
	OR	(95% CI)	OR	(95% CI)	OR	(95% CI)	OR	(95% CI)	OR	(95% CI)	OR	(95% CI)
How much does your illness affect your life?	0.98	(0.90–1.07)	0.99	(0.89–1.08)	0.96	(0.88–1.05)	0.96	(0.88–1.05)	0.96	(0.88–1.05)	0.96	(0.88–1.05)
How long do you think your illness will continue?	0.96	(0.87–1.06)	0.97	(0.88–1.07)	0.96	(0.87–1.05)	0.98	(0.89–1.08)	0.96	(0.87–1.05)	0.98	(0.89–1.08)
How much control do you feel you have over your illness?	0.94	(0.85–1.04)	0.95	(0.85–1.06)	0.98	(0.89–1.08)	0.98	(0.88–1.08)	0.98	(0.89–1.08)	0.98	(0.88–1.08)
How much do you think your treatment can help your illness?	0.99	(0.89–1.10)	1	(0.89–1.12)	0.96	(0.86–1.07)	0.96	(0.85–1.07)	0.96	(0.86–1.07)	0.96	(0.85–1.07)
How much do you experience symptoms from your illness?	1.04	(0.95–1.13)	1.03	(0.94–1.13)	1.01	(0.93–1.10)	1.01	(0.92–1.11)	1.01	(0.93–1.10)	1.01	(0.92–1.11)
How concerned are you about your illness?	1.02	(0.95–1.10)	1.03	(0.96–1.11)	0.99	(0.93–1.07)	1.01	(0.94–1.09)	0.99	(0.93–1.07)	1.01	(0.94–1.09)
How well do you feel you understand your illness?	1.05	(0.97–1.14)	1.05	(0.96–1.14)	1.06	(0.98–1.15)	0.98	(0.97–1.15)	1.06	(0.98–1.15)	1.05	(0.97–1.15)
How much does your illness affect you emotionally?(e.g. does it make you angry, scared, upset or depressed?)	1.03	(0.97–1.11)	1.05	(0.98–1.13)	1.03	(0.96–1.10)	1.03	(0.96–1.11)	1.03	(0.96–1.10)	1.03	(0.96–1.11)

*OR* odds ratio, *CI* confidence interval

^a^ Adjusted for age, education and income

Whereas beliefs about medication were not associated with poor treatment adherence on the MARS 10-item scale, several questions about necessity or concerns related to pharmaceutical treatment were able to predict poor adherence according to the Morisky scale (Table 5).

**Table 5:** Associations between medications believes and poor treatment adherence measured using three different scores in asthma patients in Latvia

	Morisky scale				MARS (5-item)				MARS (10-item)			
	Unadjusted		Adjusted^a^		Unadjusted		Adjusted		Unadjusted		Adjusted	
	OR	(95% CI)	OR	(95% CI)	OR	(95% CI)	OR	(95% CI)	OR	(95% CI)	OR	(95% CI)
Necessity												
My health is fully dependent on the asthma medication	0.67	(0.41–1.10)	0.67	(0.40–1.12)	0.58	(0.36–0.94)	0.61	(0.37–1.01)	1.07	(0.67–1.73)	1.03	(0.63–1.70)
Without asthma medication my life would be impossible	0.53	(0.33–0.85)	0.54	(0.33–0.89)	0.64	(0.41–1.01)	0.62	(0.38–1.01)	0.99	(0.62–1.55)	0.92	(0.56–1.50)
Without my asthma medication I would be very ill	0.59	(0.36–0.96)	0.61	(0.36–1.02)	0.71	(0.45–1.12)	0.72	(0.44–1.19)	0.88	(0.55–1.41)	0.85	(0.52–1.40)
My future health depends on my asthma medication	0.53	(0.31–0.90)	0.56	(0.32–0.97)	0.42	(0.25–0.70)	0.42	(0.24–0.74)	0.64	(0.38–1.07)	0.58	(0.34–1.01)
Controlling my asthma medication prevents health deteriation	0.58	(0.30–1.09)	0.66	(0.34–1.27)	0.51	(0.28–0.96)	0.61	(0.32–1.17)	0.53	(0.28–1.02)	0.53	(0.27–1.05)
Concerns												
I am concerned by the need to constantly use my asthma medication	2.03	(1.24–3.30)	1.96	(1.19–3.24)	1.69	(1.07–2.68)	1.59	(0.98–2.57)	1.36	(0.84–2.18)	1.33	(0.82–2.16)
I am sometimes concerned by long-term effects of my asthma medication	2.3	(0.27–0.71)	2.43	(1.45–4.08)	1.89	(1.19–3.01)	2	(1.22–3.27)	1.44	(0.89–2.31)	1.48	(0.90–2.42)
My asthma medication is incomprehensible to me	2.01	(1.06–3.78)	1.97	(1.02–3.80)	1.03	(0.60–1.79)	1.27	(0.70–2.29)	0.86	(0.49–1.50)	0.81	(0.45–1.46)

*OR* odds ratio, *CI* confidence interval

^a^ adjusted for age, education and income

If the patient felt that without his asthma medication his/her life would be impossible, his risk to have poor treatment adherence was 46% reduced (OR 0.54; 95% CI 0.33–0.89). Furthermore, asthma patients who were convinced that their health depends on the asthma treatment were less likely to have poor treatment adherence (OR 0.56: 95% CI 0.32–0.97). Each of the three concerns about medication questions was a statistically significant predictor of poor treatment adherence. In case the patient was concerned by the need to constantly use asthma medication or sometimes concerned by long-term effects of their asthma medication the odds of poor treatment adherence were 1.96 (95% CI 1.19–3.24) and 2.43 (95% CI 1.45–4.08), respectively. Furthermore, patients who felt that their asthma medication is incomprehensible to them had a two-fold increase in risk of poor treatment adherence. In regard MARS 5-item scale, only two variables were able to predict poor treatment adherence. The risk of poor adherence was 58% reduced (95% CI 0.24–0.74) if the patients felt that their future health depends on their asthma medication. In addition, concerns about long-term effects of asthma medication increased the risk of poor treatment adherence two-fold (95% CI 1.22–3.27).

## Discussion

### Main findings

This study found that socio-economic, socio-demographic or illness perceptions were not associated with poor asthma treatment adherence in Latvia patients. However, we revealed that concerns about the use of asthma medication and several beliefs about the necessity of asthma medication were able to predict poor treatment adherence when assessed by the Morisky or MARS 5-item adherence scales. Finally, the high prevalence of poor asthma control and treatment adherence in Latvian asthma patients is a great concern.

### Interpretation of findings in relation to previously published work

Our findings revealing that two out of three Latvian asthma patients have poor disease control are in line with previous findings indicating a prevalence of 49–76% of poor asthma control in the European population.^14–18^ Furthermore, good asthma control seemed to be more difficult to achieve in asthmatic adults who had used corticosteroids (ICS) in the last 12 months compared with those who were not in need of inhaled corticosteroids.^15^ The three treatment adherence scales used in this study revealed that close to seven out of ten asthma patients in Latvia have poor treatment adherence. Unlike poor asthma control, the prevalence of poor adherence in Latvian is considerably higher than reported in other European patient populations.^19–21^ For instance, poor adherence to asthma medication was found to be between 30–40% in France, Germany, and Italy.^19^

The health belief model tries to explain how beliefs about medicines affect ones behavior to take medication proposing that a patient chooses a certain behavior through a cost-benefit analysis where the perceived benefits such as health improvements are compared against the perceived costs.^22, 23^ Unclear results have been published about whether necessity, concerns and the necessity-concern differential correlate with adherence in asthma patients.^24–27^ In contrast to our results, some studies assessing the associations of necessity, concerns and medication adherence in asthma patients revealed that only necessity beliefs were associated to medication adherence.^24, 25^

Byer et al. found that self-reported adherence was significantly positively correlated with the necessity in 64 asthma patients were from a general practice in Leicester, UK.^24^ In addition, unlike our results they also reported a correlation between illness perception and medical adherence. Another study in 93 patients aged 18–80 years who filled at least two ICS prescriptions showed that necessities were positively related to self-reported adherence suggesting that it could be more important to focus on necessities than on concerns in an attempt to improve adherence.^25^ In line with our results, other studies Menckeberg et al. showed a positive relationship between the necessity-concerns differential and adherence indicating that having stronger beliefs about the necessity of treatment compared to concerns about negative consequences may increase adherence.^26, 27^

A possible explanation of the contradictory results may be related to the power of the study. Most of the previous studies had a much lower sample size lacking power to detect an association between concerns and medical adherence given that most likely the association between necessity and medical adherence may be stronger than for concerns and adherence. A recent meta-analysis suggested that necessity and concern beliefs about medicines are one important factor to consider when understanding reasons for non-adherence in chronic disease patients.^28^ Furthermore, they highlighted that the effect size for necessity was stronger in asthma and weaker in the cardiovascular group compared to the overall effect size. However, this may also reflect the difference in symptomatic vs. asymptomatic conditions of the diseases. An earlier meta-analysis by Horne et al. found a statistical significant association between necessity and concern beliefs and medication adherence even when stratified by country, sample size and type of adherence measure used.^29^

It was of surprise that the combined use of corticosteroids and beta 2 mimetics using the MARS (5-item) showed a lower adherence than corticosteroids as it would have been expected that the clinical effect of combination therapy would favor adherence. As this finding was only observed in the MARS (5-item) and not in the Morisky Medication Adherence Scale (MMAS) or MARS (1-item), we hypothesize that the MARS (5-item) may be confounded by the additional elements/dimensions included in the other two scales.

There are many self-report scales for measuring medication adherence. Due to the different nature of the diseases, there is no gold standard scale for measuring medication adherence.^30^ Our study showed the best associations between the necessity-concern framework with the Morisky scale but not with the MARS scale. The MARS scale has revealed adequate reliability but its validity seems to be only moderate-weak.^30, 31^ It has been suggested that though the internal consistency of the MARS could be improved either by adding more response options or by adding more items, it is debatable whether this would constitute an improvement to the measure, or whether it would compromise its quick, simple format.^30^ According to our results, we suggest to use the Morisky scale in Latvian asthma patients as a tool to assess medical adherence.

### Strengths and limitations of this study

Our study had several limitations that need to be considered. Asthma medication adherence was measured by self-report questionnaires not previously validated in the Latvian population. Even though the MMAS and the MARS 10-item scales have been successfully validated in other studies,^32–35^ self-reported measurements of medication adherence may not be that precise and still subject to self-presentational and recall bias overestimating adherence. However, the MMAS and the MARS 10-item scales have been successfully validated in other studies.^32–35^ Furthermore, asthma control was measured by a self-administered questionnaire. Clinical measurements of asthma control may be more precise and objective. However, the asthma control test (ACT) has been successfully validated in other studies consisting of a similar population than ours and has shown to be well correlated with baseline percent predicted forced expiratory volume.^36^ Thus, the ACT may correlate well with forced expiratory volume in the Latvian population as well. Furthermore, even though the patients of this study were recruited from the main towns and the capital city where the majority of the Latvian population live, the results cannot be generalized to the overall population of Latvian asthma patients. Moreover, it has to be mentioned that a general issue not addressed by self-reported adherence questionnaires is inhaler technique which is an essential part of good adherence implementation (14). The challenges faced when using inhaled medications can include a combination of delivery issues (e.g., knowing the sequence of steps required to use the inhaler correctly, successful dose preparation, inspiratory flow rate, and in pressurized metered-dose inhalers, dose actuation and coordination with breath inhalation); practical issues (e.g., integration and scheduling with coexisting medications, storage, and device cleaning); and psychosocial challenges (e.g., self-consciousness about inhaler use in public).^14, 37, 38^ Thus, it may be worthwhile developing and validating treatment adherence questionnaires that consider these important factors. Finally, the findings of our study may only be valid for a more severe patient population: those patients referred to a pulmonologist and, thus, do not necessary represent those seen in regular primary health-care. These patients may already have some issues in regard adherence as it is the most common cause of control.

### Implications for future research, policy and practice

Moreover, future studies are needed to validate the different asthma adherence scores, asthma control scores and other relevant self-assessment tools in a larger sample of Latvian asthma patients to improve their performance and utility.

## Conclusions

In conclusion, none of the tested asthma treatment adherence scales were able to predict poor asthma control in Latvian patients. However, medication beliefs, particularly concerns and necessity of asthma treatment were associated with poor treatment adherence when assessed with the Morisky or 5-item MARS scale. Therefore, we recommend to use either the MMAS or the 5-item MARS scale in Latvian asthma patients to identify patients with poor treatment adherence. Thus, it may be worthwhile to assess routinely the concerns and necessity of asthma medication in patients within the Latvian health-care system to improve treatment adherence in that vulnerable population group to improve treatment outcomes.

## Methods

### Study population

The study population of this cross-sectional patient survey consisted of asthma patients attending outpatient pulmonologist consultations in Riga, Latvia during September 2013 to December 2015. Latvian patients in most cases will receive their initial disease diagnosis and treatment initiation in a pulmonologist practice. They get there in larger part by a referral by a general practitioner (GP) or by direct patient contact. The majority of GPs refer their asthma and chronic obstructive pulmonary disease patients to a specialist at least once a year for control. The role of GPs within the Latvian health care system is mainly to ensure that a patient follows the treatment regime set forth by the specialist. Only patients referred by a GP to a National Health Service (NHS) registered practice can get their medication reimbursed by NHS. In a first step, a list of all pulmonologists from the database of the NHS of the medical doctors that have contractual rights to prescribe reimbursed medicines was acquired. Then, pulmonologists in large medical centers and hospitals in Riga and in bigger towns of Latvia were randomly selected and invited to join the study. The total number of participating pulmonologist practices was 15. Each pulmonologist was advised to invite his patients to join the survey. The sample size was calculated to detect a prevalence of poor asthma control of 50% with a margin of error of 5%, and a power of 95%. The total sample size needed and respectively studied was 352 people. Only patients that have been using asthma medication for at least one year were included in this study.

### Assessment of main variables

A self-administered questionnaire was used to assess socio-demographic and economic factors such as age, education, income, and sex. The patients filled in the questionnaire right after their visit with the doctor.

Adherence to asthma medication was assessed using MMAS and two different versions of MARS. The MMAS is a self-report tool, it was used to assess asthma medication adherence.^7^ The MMAS is an eight item questionnaire that measures medication compliance on a scale of 0–11, with lower scores indicating greater adherence. The total MMAS score was obtained by summing ratings for all scale items. Seven items were answered by either yes (score = 1) or no (score = 0) responses, and one item was assessed using 5 point Likert-type responses ranging from “usually” to “all the time” (usually = 0; all the time = 4). The MMAS has been used across many chronic diseases, including asthma, as a self-reported measure of adherence to medications and has demonstrated good reliability and predictive validity.^32, 33^ The long version of the MARS is a validated 10-item questionnaire that has shown to have good internal, construct, and criterion validity, including correlations with objective measures of adherence (electronic monitoring and pharmacy dispensing data).^8, 26^ The MARS contains items that measure intentional (“I avoid using it if I can”) and unintentional (“I forget to use it”) nonadherence and these questions are phrased such that nonadherence is considered common to minimize social desirability bias. Medication use is rated on a 5-point Likert scale.^34, 35^ The short version of the MARS is a five-item self-report scale for assessment of adherent behavior that includes assessment of unintentional non-adherent behavior (“I forgot to take them”, item 1) and intentional non-adherent behavior (“I alter the dose”, item 2. “I stop taking them for a while”, item 3. “I decide to miss out a dose”, item 4. “I take less than instructed”, item 5). Each item was answered using a five-graded response scale, ranging from very often (1) to never (5). Low scores indicate low levels of adherent behavior.^36^

Good asthma control was assessed using the ACT, a validated five-item scale that reliably assesses asthma control over a recall period of 4 weeks. The ACT consists of the following questions: “How much of the time did your asthma keep you from getting as much done at work, school or at home?”, “How often have you had shortness of breath?”, “How often did your asthma symptoms wake you up at night or earlier than usual in the morning?”, “How often have you used your rescue inhaler or nebulizer medication?” and “How would you rate your asthma control?”.^39^ Each item was scaled from 1 to 5, and by summing the response values a scale score was calculated ranging from poor (5) to total (25) control.^40, 41^ ACT scores have shown to be well correlated with baseline percent predicted forced expiratory volume.^41^

Medication beliefs were assessed using the five items of greatest relevance to asthma medication adapted from the beliefs about medicines questionnaire (BMQ), a validated tool across many disease conditions.^22, 42^ The specific–necessity scale contains 5 items that assess patients’ beliefs about specific necessity to take prescribed chronic medications. Three questions were selected from the six original items that assess patients’ beliefs about specific necessity to take prescribed chronic medications. All belief items had Likert scale responses.

The Brief IPQ was used to obtain information on illness perception of the study participants. The Brief IPQ consists of eight items and a causal question.^43, 44^ All of the items except the causal question are rated using a 0-to-10 response scale. Five of the items assess cognitive illness representations: consequences (item 1), timeline (item 2), personal control (item 3), treatment control (item 4), and identity (item 5). Two of the items assess emotional representations: concern (item 6) and emotions (item 8). One item assesses illness comprehensibility (item 7). Assessment of the causal representation is by an open-ended response, which asks patients to list the three most important causal factors in their illness (item 9).

### Statistical analysis

The Statistical Package for the Social Sciences (SPSS) IBM 21.0 was used to analyze the data. Means, standard deviations, and frequencies are presented to describe the characteristics of the study sample. A cutoff point of >6 (MARS-5), >14 (MARS-10), >1 (MMAS) was used to define poor medical adherence. These cut-off points were chosen according to the ones proposed in previous studies.^7, 8, 26, 34–36^ A cut-off point of ≤19 for the ACT was defined to indicate poorly controlled asthma, and scores of 20 points or more corresponded to well-controlled asthma.^40, 41^ The answers of the BMQ were dichotomized into (i) “I agree/I completely agree” and (ii) “Not sure/I disagree/I completely disagree”. The logistic regression analysis were first conducted for each variable alone. In the multivariate logistic analysis the outcome variable was controlled for age, income, and educational level. The odds ratio and respective 95% CI are presented for all models. The validity of each logistic regression models were assessed by the Hosmer/Lemeshow test.

### Ethics statement

Methods were performed in accordance with relevant guidelines and regulations. Methods and patient consent form were approved in writing by the P.Stradins Clinical University Hospital Development Society Ethics committee for clinical research (original name in Latvian: *P.Stradiņa Klīniskās universitātes slimnīcas Attīstības biedrības klīniskās izpētes ētikas komiteja*). A written consent was obtained from all participants involved in the study.

### Data availability

Upon request from the corresponding author.

## References

[1] Reddel HK (2015). A summary of the new GINA strategy: a roadmap to asthma control. Eur. Respir. J..

[2] Bousquet J (2010). Uniform definition of asthma severity, control, and exacerbations: document presented for the World Health Organization Consultation on Severe Asthma. J. Allergy Clin. Immunol..

[3] To T (2012). Global asthma prevalence in adults: findings from the cross-sectional world health survey. BMC Public Health.

[4] Lai CK (2009). Global variation in the prevalence and severity of asthma symptoms: phase three of the international study of asthma and allergies in childhood (ISAAC). Thorax.

[5] Bender BG, Pedan A, Varasteh LT (2016). Adherence and persistence with fluticasone propionate/salmeterol combination therapy. J. Allergy Clin. Immunol..

[6] Williams LK (2004). Relationship between adherence to inhaled corticosteroids and poor outcomes among adults with asthma. J. Allergy Clin. Immunol..

[7] Morisky D, Green L, Levine D (1986). Concurrent and predictive validity of a self-reported measure of medication adherence. Med. Care.

[8] Cohen JL (2009). Assessing the validity of self-reported medication adherence among inner-city asthmatic adults: the medication adherence report scale for asthma. Ann. Allergy Asthma Immunol..

[9] Olszanecka-Glinianowicz M, Almgren-Rachtan A (2014). The adherence and illness perception of patients diagnosed with asthma or chronic obstructive pulmonary disease treated with polytherapy using new generation cyclohaler. Postepy Dermatol Alergol.

[10] Restrepo RD (2009). Medication adherence issues in patients treated for COPD. Int. J. Chron. Obstruct. Pulmon. Dis..

[11] Unni E, Shiyanbola OO (2016). Clustering medication adherence behavior based on beliefs in medicines and illness perceptions in patients taking asthma maintenance medications. Curr. Med. Res. Opin..

[12] Chiu KC (2014). Patients’ beliefs and behaviors related to treatment adherence in patients with asthma requiring maintenance treatment in Asia. J. Asthma.

[13] Horne R, Weinman J (2002). Self-regulation and self management in asthma: exploring the role of illness perceptions and treatment beliefs in explaining non-adherence to preventer medication. Psychol. Health.

[14] Vrijens B (2016). What we mean when we talk about adherence in respiratory medicine. J. Allergy Clin. Immunol. Pract..

[15] Fueyo A, Ruiz MA, Ancochea J, Guilera M, Badia X (2007). Asthma control in Spain. Do season and treatment pattern matter? The ESCASE study. Respir. Med..

[16] Cazzoletti L (2007). Asthma control in Europe: a real-world evaluation based on an international population-based study. J. Allergy Clin. Immunol..

[17] Benkheder A (2009). Control of asthma maghreb: results of the AIRMAGstudy. Respir. Med..

[18] Rabe KF (2004). Worldwide severity and control of asthma in children and adults: the global asthma insights and reality surveys. J. Allergy Clin. Immunol..

[19] Rabe KF, Vermeire PA, Soriano JB, Maier WC (2000). Clinical management of asthma in 1999: the asthma insights and reality in Europe (AIRE) study. Eur. Respir. J..

[20] Cerveri I (1999). International variations in asthma treatment compliance: the results of the European community respiratory health survey (ECRHS). Eur. Respir. J..

[21] Bender BG, Bender SE (2005). Patient-identified barriers to asthma treatment adherence: responses to interviews, focus groups, and questionnaires. Immunol. Allergy Clin. North Am..

[22] Bender BG, Long A, Parasuraman B, Tran ZV (2007). Factors influencing patient decisions about the use of asthma controller medication. Ann. Allergy Asthma Immunol..

[23] Horne R, Weinman J (1999). Patients’ beliefs about prescribed medicines and their role in adherence to treatment in chronicphysical illness. J. Psychosom. Res..

[24] Janz NK, Becker MH (1984). The health belief model: a decade later. Health Educ. Behav..

[25] Byer B, Myers LB (2000). Psychological correlates of adherence to medication in asthma. Psychol. Health Med..

[26] Van Steenis M (2014). Relationship between medication beliefs, self-reported and refill adherence, and symptoms in patients with asthma using inhaled corticosteroids. Patient Prefer. Adherence.

[27] Menckeberg TT (2008). Beliefs about medicines predict refill adherence to inhaled corticosteroids. J. Psychosom. Res..

[28] Emilsson M (2011). The influence of personality traits and beliefs about medicines on adherence to asthma treatment. Prim. Care Respir. J..

[29] Foot H, La Caze A, Gujral G, Cottrell N (2016). The necessity-concerns framework predicts adherence to medication in multiple illness conditions: a meta-analysis. Patient Educ. Couns..

[30] Horne SC (2013). Understanding analytic review of the necessity–concerns framework. PloS ONE.

[31] Culig J, Leppée M (2014). From Morisky to Hill-bone; self-reports scales for measuring adherence to medication. Coll. Antropol..

[32] Thompson K, Kulkarni J, Sergejew AA (2000). Reliability and validity of a new medication adherence rating scale (MARS) for the psychoses. Schizophr. Res..

[33] Krousel-Wood M, Thomas S, Muntner P, Morisky D (2004). Medication adherence: a key factor in achieving blood pressure control and good clinical outcomes in hypertensive patients. Curr. Opin. Cardiol..

[34] Krapek K (2004). Medication adherence and associated hemoglobin A1c in Type 2 diabetes. Ann. Pharmacother..

[35] Cochrane GM, Horne R, Chanez P (1999). Compliance in asthma. Respir. Med..

[36] Wroe AL (2002). Intentional and unintentional nonadherence: a study of decision making. J. Behav. Med..

[37] Westerik JA (2016). Characteristics of patients making serious inhaler errors with a dry powder inhaler and association with asthma-related events in a primary care setting. J. Asthma.

[38] Giraud V, Roche N (2002). Misuse of corticosteroid metered-dose inhaler is associated with decreased asthma stability. Eur. Respir. J..

[39] Horne R., & Hankins M. (eds) *The Medication Adherence Report Scale (MARS)*. (University of Brighton, 2004).

[40] Nathan RA (2004). Development of the asthma control test: a survey for assessing asthma control. J. Allergy Clin. Immunol..

[41] Schatz M (2006). Asthma control test: reliability, validity, and responsiveness in patients not previously followed by asthma specialists. J. Allergy Clin. Immunol..

[42] Horne R, Weinman J, Hankins M (1999). The beliefs about medicines questionnaire: the development and evaluation of a new method for assessing the cognitive representation of medication. Psychol. Health.

[43] Weinman J, Petrie KJ, Moss-Morris R, Horne R (1996). The illness perception questionnaire: a new method for assessing the cognitive representation of illness. Psychol. Health.

[44] Moss-Morris R (2002). The revised illness perception questionnaire (IPQ-R). Psychol. Health.

